# Intermittent Fasting: A Metabolically Focused Therapeutic Strategy for Obesity

**DOI:** 10.3390/nu18030371

**Published:** 2026-01-23

**Authors:** Natalia Diaz-Garrido, Sebastián Zagmutt, Alejandro Regaldiz, Pedro Cisternas, Marianela Bastías-Pérez

**Affiliations:** 1 Universidad Católica del Maule, Facultad de Ciencias de la Salud, Escuela de Nutrición y Dietética, Curicó 3340000, Chile; npdiaz@ucm.cl; 2 Department of Biomedical Sciences, Faculty of Medicine and Health Sciences, Universitat Internacional de Catalunya, 08195 Sant Cugat del Vallès, Spain; szagmutt@uic.es; 3 Programa de Doctorado en Biociencias Moleculares, Facultad de Ciencias de la Vida, Universidad Andres Bello, Santiago 8370186, Chile; regaldis.alejandro@gmail.com; 4 Núcleo de Investigación en Nutrición y Ciencias Alimentarias (NINCAL), Facultad de Salud y Ciencias Sociales, Universidad de Las Américas, Santiago 7500658, Chile; pcisternasf@udla.cl; 5 Centro de Investigación en Ciencias Biológicas y Químicas (CICBQ), Facultad de Medicina Veterinaria y Agronomía, Universidad de Las Américas, Santiago 7500658, Chile; 6 Centro de Investigación Biomédica en Red (CIBER) de Fisiopatología de la Obesidad y Nutrición (CIBEROBN), Instituto de Salud Carlos III (ISCIII), E-28029 Madrid, Spain

**Keywords:** intermittent fasting (IF), alternate day fasting (ADF), time-restricted eating (TRE), thermogenesis, brown adipose tissue (BAT), CNS-BAT communication, browning

## Abstract

The global prevalence of obesity continues to rise and is a significant risk factor for the onset and progression of cardiovascular diseases. Despite the development of new pharmacological therapies, novel strategies are being explored to mitigate the impact of this disease. Intermittent fasting (IF) is a nutritional intervention that has gained popularity and shows potential as an innovative approach to weight management. This study aims to compile scientific evidence on various aspects of fasting, including its physiological effects, the molecular and thermogenic mechanisms involved, and recommendations regarding nutritional strategies during the refeeding period within the eating window. We conducted a narrative review, analyzing evidence available from PubMed/MEDLINE based on studies related to intermittent fasting, thermogenesis, and their associated outcomes. Our results demonstrate the existence of three commonly used IF protocols: alternate day fasting (ADF), periodic fasting (PF), and time-restricted eating (TRE). In addition to its effects on weight loss, IF has demonstrated notable benefits for cardiovascular health, oxidative stress, and metabolic function. Moreover, the interaction between the central nervous system and brown adipose tissue provides an alternative mechanism for the molecular regulation of thermogenesis. Nutritional patterns adopted during intermittent fasting play a crucial role in optimizing outcomes, with particular emphasis on the intake of proteins, fiber, bioactive compounds, and essential fatty acids during the feeding window. In summary, current evidence indicates that intermittent fasting provides a biologically robust framework for studying energy balance and holds promise for developing targeted nutritional interventions.

## 1. Introduction

In recent decades, the global prevalence of obesity has substantially increased. Currently, an estimated 650 million adults and approximately 340 million children and adolescents aged 5–19 years are affected by obesity worldwide [1]. This condition arises from an imbalance between caloric intake and energy expenditure, leading to excessive accumulation of adipose tissue as a consequence of a sustained positive energy balance [2]. Obesity is associated with significant health risks, including type 2 diabetes mellitus (T2DM) [3], dyslipidemias [4], and various types of cancer [5], all of which are linked to insulin resistance and lipotoxicity [6]. In addition to genetic and environmental factors, various pathological cellular processes, such as chronic inflammation and mitochondrial dysfunction, contribute to the development and progression of the disease [7,8].

Although there have been remarkable advancements in pharmacological therapeutic interventions based on pharmacology [9], there has been increasing interest in improving dietary patterns through dietary interventions [10]. In this regard, intermittent fasting (IF) appears to be one of the most popular strategies for controlling caloric intake, showing benefits in weight management, cardiovascular health, oxidative stress, and general health and lifestyle outcomes [11]. IF is a dietary pattern that cycles between fasting periods and regular ad libitum eating on a scheduled basis, during which individuals severely restrict their caloric intake within a day or week (Table 1) [12,13]. It has been suggested that IF is less restrictive, simpler, and more flexible than traditional calorie restriction (CR) methods [14]. Studies in humans have reported several health benefits, including reductions in fat mass and body weight, improvements in glucose tolerance and insulin sensitivity, and lower risks of cardiovascular disease and cancer [12,14,15]. However, more evidence is needed to evaluate the risks, benefits, and adherence to IF, particularly in individuals with obesity [12,15,16,17,18]. Likewise, studies in animals have shown that IF can modify adipose tissue morphology and induce changes in tissue growth dynamics [19]. Notably, sexual dimorphisms in IF responses remain an important research gap, given the significant variations in inflammatory processes and diet-induced obesity between men and women [20,21].

IF induces long-term “metabolic adaptation”, which can reduce the metabolic rate and may extend human lifespan [22]. In this context, during IF, a metabolic switch is activated, shifting energy utilization from glucose to fatty acids and ketone bodies, which represents an evolutionary mechanism that reprograms metabolism [23]. Therefore, IF may provide a targeted strategy for restoring metabolic flexibility, improving insulin sensitivity, and supporting healthy body composition [13]. In addition, the ability of IF to modulate glucoregulatory factors has been frequently assessed in clinical studies. In particular, subjects with T2DM have shown significant beneficial effects of IF in both diabetic animal models and T2DM individuals [24].

Therefore, this review aims to compile scientific information on the different IF schemes and their effects. In addition, the molecular mechanisms involved in IF, including those from the perspectives of neuroscience and thermogenesis, are discussed. Finally, some alternatives for refeeding during the eating window are reviewed, highlighting the use of diet-induced thermogenesis (DIT) as a novel nutritional strategy.

**Table 1:** Approaches and characteristics of intermittent fasting.

Features	Alternate-Day Fasting (ADF)	Periodic Fasting (PF)	Time-Restricted Eating (TRE)	References
**Principle**	Compensatory Metabolic Responses	Compensatory Metabolic Responses	Circadian Rhythms	[25]
**Frequency**	Every other day	Two days weekly	Every day	[11]
**Duration** **(fasting period)**	24 h	24 h each fasting day	14–18 h	[26]
**Eating window**	24 h (ad libitum on feeding days)	24 h (ad libitum on feeding days)	4–12 h	[27]
**Energy intake during fasting periods**	Strictly controlled energy intake (≈25% of usual intake, ≈500 kcal)	Strictly controlled energy intake (500–800 kcal)	No energy intake (water allowed)	[23]

## 2. Literature Search Methods

A structured narrative search was conducted in the PubMed/MEDLINE database up to October 2025. The search strategy employed MeSH terms and free-text terms combined with the Boolean operators OR and AND, including “intermittent fasting”, “time-restricted eating”, “alternate-day fasting”, “thermogenesis”, “brown adipose tissue”, “white adipose tissue”, “CNS–BAT communication”, and “browning”. Original preclinical and clinical studies, recent systematic reviews and meta-analyses, and relevant in vitro and in vivo studies related to intermittent fasting, thermogenesis, and associated outcomes were included. Editorial letters, non-peer-reviewed sources, and studies not published in English were excluded. Reference lists of key articles were also hand-searched. Owing to the substantial heterogeneity in study designs and reported outcomes, a narrative synthesis was undertaken, focusing on the clinical outcomes and molecular mechanisms related to thermogenesis.

## 3. Intermittent Fasting

**Figure 1:**
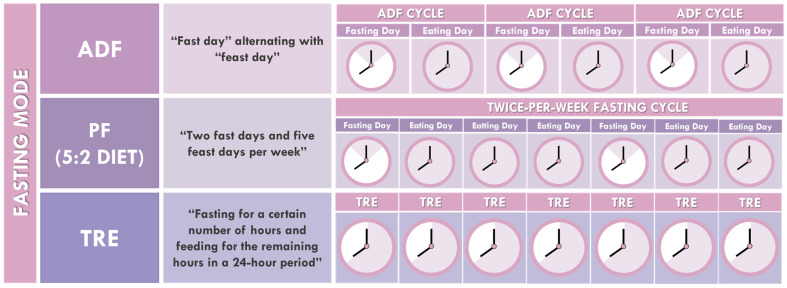
Types of intermittent fasting. Three commonly used IF protocols are illustrated: alternate-day fasting (ADF), periodic fasting (5:2), and time-restricted eating (TRE). The diagrams depict the fasting duration and corresponding eating windows for each approach. Darker shading indicates eating periods within the fasting regimen, facilitating visual comparisons of timing and duration across protocols.

IF is an innovative approach to weight control, consisting of different timing schedules for temporary food avoidance in healthy individuals. Its particularity relies on producing favorable metabolic effects by intermittently shifting the metabolism from fatty acids to ketones. Three types of IF have received the most research attention: Alternate-day fasting (ADF), periodic fasting (5:2 diet) and time-restricted eating (TRE) (Figure 1).

ADF involves a “fast day” alternating with a “feast day”, with fasting for 24 h every other day [26]. On feast days, individuals are allowed to eat ad libitum, with no restrictions on the types or quantities of food consumed. During fasting days, individuals may choose to consume only water, which is referred to as “zero-calorie alternate-day fasting” [11]. Alternatively, individuals can consume 25% of their energy needs (500–800 kcal per day), which is known as “modified alternate-day fasting” [28]. Similarly, PF is a modified version of ADF that includes two fast days and five feast days per week (5:2 diet) [29]. Fasting can occur on consecutive or nonconsecutive days, and individuals are permitted to consume 25% of their energy needs (500–1000 kcal per day). Likewise, on feast days, individuals are allowed to eat ad libitum [30]. Finally, the TRE protocol involves a daily routine that requires fasting for a specific number of hours and feeding during the remaining hours of a 24-h period [17]. TRE typically includes an eating window of 4–8 h, with fasting supported by water or zero-calorie beverages for the rest of the day. The most commonly used protocol is 16/8 [24].

### 3.1. Effects of Intermittent Fasting

#### 3.1.1. Corporal Weight and Body Composition

IF appears to be an effective method that may be beneficial for obesity [25,31]. Animal studies and human clinical trials have reported that the main outcome of IF is weight loss, but it also impacts visceral obesity, cardiometabolic health, metabolism, and glucose homeostasis [28,32,33]. Most human clinical trials have been conducted on normal-weight, overweight, and obese individuals [34,35,36,37,38,39,40,41,42,43,44,45]. In particular, ADF and the 5:2 diet produce similar degrees of weight loss (4–8% from baseline) in men and women with obesity over short intervention periods of 8–12 weeks [28,34,40,41,42,46,47,48]. Studies of longer duration (12–52 weeks) have not shown greater reductions in body weight, suggesting that weight loss efficacy may peak around 12 weeks, with reductions of 0.2–0.5 kg per week [36,37,43,44,49]. Compared with ADF and the 5:2 diet, the TRE regimen has shown a less marked degree of weight loss, approximately 3–4% from baseline [38,39,50]. Notably, both ADF and the 5:2 diet helped prevent weight regain during the 12–24 months of follow-up [34,35,37,43]. When ADF and 5:2 are implemented as maintenance diets, the energy intake allowed on fast days is increased (1000–1200 kcal per day instead of 500 kcal/day), which facilitates weight maintenance. Regarding body composition, during IF practices, most of the weight lost corresponds to a reduction in fat mass. No differences have been reported between IF and continuous calorie restriction in terms of the type of mass loss [51,52,53,54]. However, it has been observed that when the TRE regimen is combined with exercise, subcutaneous fat mass decreases significantly [17,55,56]. Remarkably, individuals following ADF or the 5:2 diet showed significant decreases in visceral fat mass compared with controls, whereas no changes were reported with the TRE regimen [34,35,36,37,40,43,49].

#### 3.1.2. Metabolic Risk Factors

*Blood pressure.* The effect of IF on blood pressure is currently controversial. While several studies have shown reductions in systolic and diastolic blood pressure in humans [40,43,45,49,57,58], others have demonstrated the opposite effects [35,36,38,41,44,48,59,60,61,62]. Notably, most trials reporting decreased blood pressure involved participants with elevated blood pressure at baseline or individuals older than 45 years [63]. Additionally, IF has been observed to decrease blood pressure in overweight or obese populations, even without weight loss [30]. One potential explanation for this effect may be associated with the activation of the parasympathetic system, driven by increased activity of cholinergic neurons and elevated venous plasma levels of noradrenaline [64,65].

*Lipid profile.* The relationship between plasma lipids and IF remains debatable [66,67]. A limitation of the current evidence is the lack of clarity regarding the timing of blood sample collection during IF protocols [26]. Several studies have shown that fasting plasma triglyceride concentrations are not affected after 4–10 h of TRE interventions [38,39,50,68,69]. Only one study reported that 9 h of TRE for 7 days led to a significant decrease in triglyceride levels, suggesting that these levels may depend on additional factors [70]. While some studies observed reductions in total cholesterol levels in overweight individuals [39,68], others found no significant effects of IF [38,68]. LDL cholesterol (LDL-c) concentrations decreased by 10–22% from baseline in three IF trials [34,37,49]. However, most studies indicate that TRE for 5–12 weeks does not affect LDL-c in individuals with excessive adiposity [38,39,50,57]. Similarly, HDL cholesterol (HDL-c) levels generally remain unchanged or slightly decrease with IF [34,37,43,49]. HDL-c concentrations may decline during the acute phase of weight loss but tend to rebound to baseline levels thereafter [71].

*Glucose homeostasis and insulin sensitivity.* Fasting plasma glucose levels do not change under IF alternatives, including ADF, the 5:2 diet, and TRE [35,41,46]. Almost all studies have been conducted in individuals without type 1 DM (T1DM) or T2DM at baseline, in whom fasting glucose is generally well controlled [72]. Additionally, fasting insulin levels were reduced from the baseline in several trials [35,37,38,48,49,57]. This pattern has been observed mostly in individuals with baseline hyperinsulinemia compared to those with normal insulin levels [37,38,40]. The modified 5:2 diet affects body weight in patients with T2DM [13,37]; however, adherence to this IF alternative is challenging. In contrast, TRE appears to be an appropriate alternative to decrease HbA1c (hemoglobin A1c) levels in individuals with diabetes, although this effect is observed only over long-term periods [13,73]. Patients with T1DM and obesity have shown changes in body weight but not in HbA1c levels [44]. IF diets do not appear to significantly affect glucose management, although the certainty of this evidence remains low [74].

*Inflammation and oxidative stress.* Evidence shows that IF decreases plasma levels of proinflammatory molecules such as tumor necrosis factor alpha (TNF-α), homocysteine, interleukin-6 (IL-6), and C-reactive protein (CRP), all of which contribute to atherosclerotic plaque formation [75]. Despite these findings, other studies suggest that IF generally does not affect these circulating biomarkers [38,50,55,57]. Moreover, IF has been shown to increase adiponectin secretion from adipocytes, which inhibits the release of the adhesion molecules VCAM and ICAM from the endothelium, resulting in reduced local and systemic inflammation [41,47,64]. In parallel, IF enhances the body’s antioxidant defense systems, as evidenced by a reduction in malondialdehyde (MDA) levels and an increase in glutathione (GSH) during fasting periods [76]. Furthermore, the upregulation of enzymes such as superoxide dismutase-2 (SOD-2) and nuclear factor erythroid 2-related factor 2 (Nrf-2) during IF reinforces antioxidant activity [77]. Other plasma markers have also shown decreased levels under IF, including 8-isoprostane, nitrotyrosine, oxidized protein products, and carbonyls [38,49,57,77,78].

Given the physiological complexity and distinct metabolic features of IF, the next section examines how this dietary pattern influences thermogenesis and the molecular mechanisms that support its effects.

## 4. Intermittent Fasting and Thermogenesis: The Role of Adipose Tissue

The primary functions of white adipose tissue (WAT) and brown adipose tissue (BAT) are energy storage and thermogenesis, respectively [79,80]. Both tissues exert endocrine functions by secreting hormones and factors that regulate energy homeostasis, glucose and lipid metabolism, and cellular inflammatory processes [80,81,82]. Differences between WAT and BAT include gene expression patterns and the origin of their progenitor cells [79]. The characteristic color of BAT results from its abundant mitochondria and multilocular lipid droplets, which support the inherent thermogenic properties of brown adipocytes in BAT depots. BAT adipocytes oxidize lipids in response to sympathetic nervous system activation and other hormonal stimuli to generate heat, thereby contributing to thermogenesis. In contrast, WAT is associated with macrophage infiltration and increased secretion of inflammatory cytokines in the context of obesity and metabolic disorders. In response to appropriate stimuli, WAT depots may transition into BAT in a process known as “browning”, which gives rise to beige adipose tissue that displays characteristics of both depots. External stimuli, such as cold exposure, exercise, fasting, and pharmacological treatments, promote the development of beige adipocytes in WAT in mice. Animal studies have shown that daily caloric restriction and IF promote WAT browning [83,84,85], possibly mediated through alternative macrophage activation. In humans, however, WAT browning has been reported mainly in response to extreme adrenergic stimulation, such as burn injuries or the administration of β3-adrenergic receptor agonists. Therefore, BAT activation and browning are promising strategies for increasing energy expenditure and combating obesity [86]. Given the central role and excessive accumulation of WAT in obesity, stimulating WAT browning through different approaches or therapies may represent an alternative to improve metabolic health and reduce obesity [87,88,89].

BAT plays a crucial role in energy homeostasis through thermogenesis [86], which is mediated by uncoupling protein 1 (UCP1) or thermogenin [90], by uncoupling mitochondrial respiration from adenosine triphosphate (ATP) synthesis, ultimately generating heat. In addition to its thermogenic function, BAT is recognized for its endocrine capacity, suggesting that it may play a key role in the regulation of systemic energetic homeostasis. Experimental evidence supports that BAT secretes regulatory molecules and utilizes metabolic substrates during thermogenesis [91]. Brown and beige adipocytes secrete autocrine and paracrine factors that modulate their own expansion and activity, as well as the browning of WAT [92]. BAT releases endocrine factors that act on various peripheral tissues, including WAT, liver, pancreas, heart, and bone, and also influences systemic metabolism through interactions with the central nervous system. These endocrine factors, termed “batokines”, include fibroblast growth factor 21 (FGF21), IL-6, and neuregulin 4. They exert beneficial metabolic effects and may be key components of therapeutic strategies to control obesity and its associated metabolic complications. BAT, as a primary site of adaptive thermogenesis, has been experimentally associated with protection against obesity and metabolic diseases such as T2DM and dyslipidemia. Although the ability of BAT to utilize glucose and lipids for thermogenesis is considered essential for heat production, its emerging endocrine role is increasingly recognized as fundamental to these protective effects.

To elucidate the link between thermogenesis and its molecular effects, it is essential to examine the underlying brain mechanisms. The following section details the central nervous system pathways and their role in regulating thermogenesis.

## 5. Brain–Brown Fat Crosstalk: Mechanisms of Thermogenic Regulation During Intermittent Fasting

The regulation of BAT thermogenesis by the central nervous system (CNS) represents a fundamental mechanism for maintaining energy balance and metabolic flexibility [93,94]. Classical studies have established that BAT activation depends on sympathetic outflow originating from the hypothalamic and brainstem circuits [95,96], whereas recent work has revealed a far more complex and bidirectional communication between the brain and peripheral metabolic signals [97,98,99]. In this context, IF provides a physiological framework to examine how the CNS dynamically recalibrates energy expenditure according to nutrient availability, although the molecular and circuit-level mechanisms remain incompletely understood.

Thermogenic control originates in hypothalamic nuclei such as the preoptic area (POA), dorsomedial hypothalamus (DMH), and paraventricular nucleus (PVH), which integrate thermal, sensory, and hormonal cues to regulate sympathetic premotor neurons in the rostral raphe pallidus (rRPa) and intermediolateral spinal nucleus (IML) [100,101,102]. Activation of this axis triggers norepinephrine release onto β3-adrenergic receptors, driving lipolysis and mitochondrial uncoupling via UCP1, although alternative pathways, such as calcium cycling, creatine phosphate cycling, and futile lipid turnover, enable thermogenesis independently of UCP1 [103]. The DMH–rRPa pathway also mediates stress-induced thermogenesis [104], whereas hypothalamic orexigenic (NPY/AgRP) and anorexigenic (POMC, orexin, and MCH) neurons contribute to diet- and hormone-dependent regulation [105]. AgRP and POMC neurons in the arcuate nucleus (ARC) are particularly sensitive to energy status, integrating leptin, insulin, nutrient, and neural signals. During fasting, declining leptin and insulin levels reduce the excitability of ARC, DMH, and PVH neurons, transiently suppressing sympathetic outflow and BAT thermogenesis [106,107]. Leptin administration reverses this inhibition, confirming its role as a permissive signal. Hypothalamic nutrient sensors, including AMPK, mTOR, and SIRT1, detect shifts in cellular energy status and modulate sympathetic drive to BAT [108,109], linking energy deprivation to adaptive reductions in thermogenesis. Zagmutt et al. demonstrated that loss of CPT1A in AgRP neurons enhances BAT activation and reduces weight gain, with a stronger effect in females, highlighting the importance of central energetic status in thermogenic regulation [21].

**Figure 2:**
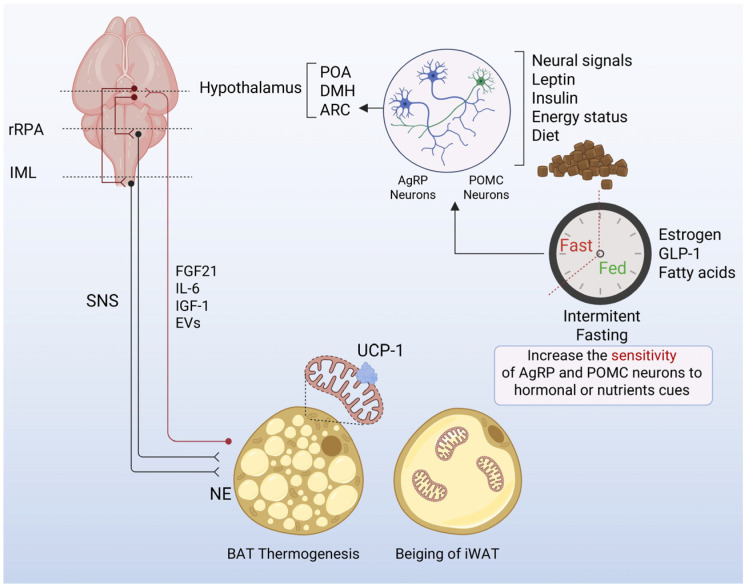
Central and peripheral mechanisms by which intermittent fasting (IF) modulates brown adipose tissue (BAT) thermogenesis. Intermittent fasting imposes recurrent cycles of fasting and refeeding, which alter the activity and hormonal sensitivity of hypothalamic circuits involved in energy balance. During fasting, reduced leptin and insulin levels suppress thermogenic output by decreasing ARC POMC neuron activity and enhancing AgRP neuronal signaling, ultimately lowering sympathetic drive to BAT. Refeeding restores hormonal cues and increases the responsiveness of these neurons, enabling rapid reinstatement of BAT thermogenesis. IF is proposed to heighten the sensitivity of AgRP and POMC neurons to metabolic and nutrient-derived signals, facilitating dynamic switching between low- and high-thermogenic states. Peripheral adaptations include shifts in lipid handling and mitochondrial remodeling in adipose depots, with potential recruitment of beige adipocytes. Together, these CNS–adipose interactions position IF as a modulator of thermogenesis and metabolic flexibility in adipose tissue. Figure was created using BioRender.com. Abbreviations: ARC, arcuate nucleus; AgRP, agouti-related peptide; POMC, proopiomelanocortin; POA, preoptic area; DMH, dorsomedial hypothalamus; BAT, brown adipose tissue; WAT, white adipose tissue; IF, intermittent fasting; NE, norepinephrine; UCP1, uncoupling protein 1; SNS, sympathetic nervous system.

IF imposes repeated cycles of energy deprivation and refeeding, which likely amplify these adaptive mechanisms. Repeated fasting–refeeding may increase the sensitivity of AgRP and POMC neurons to hormonal cues, promoting the efficient suppression of BAT thermogenesis during fasting and rapid rebound upon refeeding. Peripheral signals, including BMP8 [110], estrogens [111], GLP-1 [112], and fatty acids [113], further refine these responses. Together, these observations suggest that IF leverages hypothalamic energy-sensing circuits to dynamically recalibrate thermogenesis, optimizing energy expenditure and metabolic flexibility under oscillating nutrient states (Figure 2).

Beyond efferent control, BAT also communicates back to the CNS via batokines, including FGF21, IL-6, and IGF-1, as well as extracellular vesicles carrying microRNAs and lipids [114,115,116]. These signals influence appetite, glucose metabolism, and autonomic tone, highlighting BAT as an active participant in systemic energy sensing rather than a mere effector (Figure 2).

IF, cold exposure, and exercise converge on transcriptional programs involving UCP1, PGC-1α, PRDM16, and SIRT3, promoting mitochondrial biogenesis and oxidative metabolism in brown and beige adipocytes [84,117,118]. Yet, how the CNS integrates these peripheral adaptations to coordinate whole-body energy expenditure remains unclear, and repeated fasting–refeeding cycles may induce long-term “metabolic memory” by altering hypothalamic sensitivity to leptin, insulin, or incretins. At the same time, the role of BAT in mediating fasting-induced thermogenesis remains controversial. Studies using ADF models have shown that this intervention selectively induces beigeing of inguinal white adipose tissue (iWAT) rather than activation of BAT, with suppressed UCP1 expression and no upregulation of other classical thermogenic genes [83].

Complementing these observations, proteomic analyses revealed that ADF increases mitochondrial protein content in both subcutaneous (scWAT) and visceral white adipose tissue (vWAT), whereas BAT remains largely unaffected. In parallel, fatty acid synthesis enzymes are upregulated in WAT depots, but lipolysis is specifically downregulated in vWAT due to decreased ADRB3 abundance, which helps preserve visceral lipid stores during fasting. Additionally, downregulation of inflammatory collagen IV in vWAT improves insulin sensitivity. Together, these findings indicate that intermittent fasting triggers depot-specific adaptations, favoring beige adipocyte recruitment and mitochondrial remodeling in WAT while classical BAT activation may be limited [119].

In contrast, other IF protocols, such as TRE (16/8 regimen), appear to directly engage BAT thermogenesis. Mice subjected to 16/8 IF showed improved glucose homeostasis, reduced body weight, food intake, and overall adiposity, along with increased postprandial oxygen consumption (VO_2_), heat production, BAT temperature, and ketone bodies. Crucially, postprandial thermogenesis is abolished after BAT denervation or Ucp1 deletion, and most metabolic effects are absent in leptin-deficient ob/ob mice, indicating that BAT activation under 16/8 IF is UCP1- and leptin-dependent. Additionally, 16/8 IF enhances leptin sensitivity, further linking hypothalamic energy-sensing circuits to BAT-mediated energy expenditure [120].

These observations collectively suggest that intermittent fasting triggers protocol-specific thermogenic adaptations: some regimens, like ADF, preferentially recruit beige adipocytes and β-adrenergic-independent pathways, whereas others, like TRE protocol, robustly activate classical BAT via UCP1 and leptin signaling (Figure 2). This highlights the complexity of CNS–adipose communication and underscores the need for further studies to delineate how different IF strategies modulate energy balance under oscillating nutrient states.

Despite recent advances, major questions remain. It is unclear whether IF sensitizes or desensitizes hypothalamic neurons to metabolic hormones, how batokines influence central networks, and the contribution of UCP1-independent thermogenesis across species. Integration with microbiome-derived metabolites, exosomal communication, and hypothalamic inflammatory signaling also warrants investigation. Translating these findings to humans, particularly regarding melanocortin and catecholaminergic regulation of BAT, remains challenging. Future studies should use temporally resolved experimental designs, combining optogenetic or chemogenetic manipulation of fasting-responsive neurons with measures of sympathetic activity, UCP1 expression, and circulating batokines. Integrative omics, including ATAC-seq and single-cell RNA-seq in hypothalamic nuclei, will be essential to reveal transcriptional and epigenetic mechanisms of adaptive thermogenesis. In humans, multimodal PET-CT imaging with hormonal and inflammatory profiling may help translate these insights clinically.

From a therapeutic perspective, targeting the brain–BAT axis represents an attractive strategy to combat obesity. However, metabolic adaptation during caloric restriction often limits thermogenic output. Combining IF with GLP-1 receptor agonists, β3-adrenergic sensitizers, or batokine-based therapies could enhance BAT recruitment if leptin-permissive signaling is maintained. Understanding CNS–BAT communication during fasting will inform strategies to safely increase energy expenditure and metabolic resilience.

To effectively implement and optimize IF, it is important to recognize that its benefits depend on specific nutritional strategies. The following section discusses and highlights food groups that can support the outcomes of IF practices.

## 6. Nutritional Strategies During Refeeding

While fasting initiates beneficial metabolic adaptations, the refeeding period plays an equally critical role in determining long-term outcomes. The timing, duration, and composition of meals consumed after fasting can either reinforce or counteract these metabolic benefits, making nutritional strategies during refeeding a central focus for optimizing IF protocols.

### 6.1. Timing and Duration of Refeeding Windows

The timing and duration of the eating window play a decisive role in sustaining the benefits of the IF-induced metabolic switch. Narrower refeeding periods favor fat oxidation, improve insulin sensitivity, and reduce postprandial spikes in blood glucose levels compared with unrestricted feeding schedules [27,121]. In addition, synchronizing refeeding with circadian rhythms further enhances these effects. Early time-restricted feeding, in which meals are consumed earlier in the day, has been associated with improved glycemic control, blood pressure, and lipid metabolism [57]. In contrast, late-night eating tends to impair glucose tolerance and may counteract fasting-induced benefits [122]. Thus, optimizing the refeeding window not only supports metabolic flexibility but also synergizes with intrinsic circadian regulation of energy metabolism.

### 6.2. Thermogenic Ingredients and Diet-Induced Thermogenesis in Refeeding Windows

Refeeding after fasting represents a critical window during which dietary choices can either reinforce or counteract fasting-induced adaptations. A key mechanism is diet-induced thermogenesis (DIT), which contributes approximately 8–10% of the total daily energy expenditure, in addition to basal metabolic rate, physical activity, and adaptive thermogenesis [123,124,125]. Because diet-induced thermogenesis varies in magnitude according to macronutrient composition (highest for protein, intermediate for carbohydrate, and lowest for fat), DIT represents a modifiable factor that can be strategically leveraged during refeeding [124].

*Protein and Fiber*. Proteins have the strongest thermogenic effect, with 20–30% of the ingested energy dissipated as heat. Clinical evidence has shown that combining IF with protein pacing results in greater reductions in total and visceral fat than continuous caloric restriction [126]. Moreover, variations in protein content during fasting-mimicking diets modulate metabolic responses, underscoring the importance of protein quality and timing during refeeding [127]. Dietary fiber further supports this metabolic switch by improving glycemic control and insulin sensitivity [128]. Fermentable fibers generate short-chain fatty acids (SCFAs) that may modestly increase thermogenesis. In overweight adults, combining fiber with probiotic supplementation increased resting energy expenditure by approximately 84 kcal/day [129]. Still, the effect of fiber depends on its type and context, as some high-fiber meals have been shown to reduce postprandial thermogenesis while increasing satiety [130,131].

*Bioactive Compounds.* Several phytochemicals enhance thermogenesis through sympathetic activation or direct stimulation of BAT. Capsaicin from chili peppers increases fat oxidation and total energy expenditure [132], and additional findings confirm that both capsaicin and grains of paradise, a species of the ginger family, can activate BAT in humans [133]. Green tea catechins and caffeine exert synergistic effects, modestly raising resting energy expenditure and fat oxidation [134].

*Lipid Quality.* The type of fat consumed during refeeding strongly influences DIT. Medium-chain triglycerides (MCTs) are rapidly oxidized and stimulate ketone production, resulting in a higher thermogenic effect compared with long-chain fats [135]. Polyunsaturated fats, particularly omega-3 fatty acids, also support mitochondrial efficiency and fat oxidation, whereas excessive saturated fat intake is associated with lower thermogenesis and impaired insulin sensitivity [136,137].

*Polyphenols and Anthocyanins.* Polyphenol-rich foods may complement fasting adaptations by modulating energy expenditure and substrate utilization. In diet-induced obese mice, maqui berry (Aristotelia chilensis), rich in anthocyanins, induced browning of WAT, improved glucose tolerance, and reduced hepatic steatosis [138,139]. These findings suggest that anthocyanin-rich foods could enhance metabolic flexibility when included in refeeding meals.

*Foods and Patterns to Limit.* Not all foods support fasting-induced benefits. Highly processed and refined foods reduce the energy cost of digestion and blunt satiety [131]. High-glycemic foods produce exaggerated postprandial glucose and insulin responses, thereby suppressing fat oxidation [140,141]. Large, late-night meals misalign feeding with circadian rhythms, lowering glucose tolerance and thermogenesis [122]. Alcohol further contributes to calories with a minimal thermogenic effect and impairs fat oxidation, promoting hepatic fat accumulation [142].

DIT can potentially be enhanced by optimizing refeeding meals, emphasizing protein, fermentable fibers, thermogenic bioactives, and beneficial fats, while limiting refined carbohydrates, saturated fats, late-night eating, and alcohol. These strategies may help sustain the metabolic switch, promote metabolic flexibility, and reinforce the physiological benefits of intermittent fasting.

## 7. Discussion

While substantial advancements have been achieved in pharmacological therapies, there has been a growing emphasis in recent years on optimizing dietary patterns through nutritional interventions [29]. Within this framework, IF has emerged as a prominent strategy for modulating caloric intake, with evidence indicating its beneficial effects on weight management, cardiovascular health, oxidative stress, and overall health and lifestyle outcomes [24].

Our results identified three primary IF protocols: alternate-day fasting (ADF), periodic fasting (PF), and time-restricted eating (TRE) [27]. Despite their documented benefits, the practicality of implementing such dietary approaches in daily life remains uncertain. One argument supporting IF is its greater adaptability compared to other restrictive dietary regimens, such as ketogenic, vegan, or daily caloric restriction diets [25]. Importantly, because IF aligns with circadian rhythms, it may represent a more physiologically compatible dietary strategy, potentially offering adaptive advantages over alternative approaches [143]. Tinsley et al. evaluated the feasibility of TRE in mitigating lifestyle-related diseases and found that TRE is not only practicable but also associated with improvements in quality of life [59]. Similarly, Wegman et al. reported that IF demonstrated high adherence rates, further supporting its feasibility [144].

However, it remains unclear whether IF affects appetite regulation. Beaulieu et al. reported that fasting did not result in a compensatory increase in appetite or appetite loss when weight loss did not exceed 5% [145]. In contrast, the Early Time-Restricted Feeding study found that TRE reduced mean ghrelin levels and decreased participants’ desire to eat [146]. Furthermore, our findings suggest that the inclusion of specific food groups during the eating window may optimize the outcomes of IF. Such dietary strategies may help sustain the metabolic switch, enhance metabolic flexibility, and reinforce the physiological benefits associated with IF [57]. In this context, diet-induced thermogenesis (DIT) is primarily driven by the intake of proteins, dietary fiber, bioactive compounds, and essential fatty acids, which are strongly recommended for breaking a fast because of their pronounced capacity to stimulate thermogenesis and modulate metabolic adaptations during the fasting state [123,124].

Thermogenesis represents a central molecular mechanism activated during fasting. Brown adipose tissue (BAT) oxidizes lipids in response to sympathetic nervous system activation and hormonal stimuli, thereby generating heat and contributing to whole-body thermogenesis [80]. Our findings indicate that, under appropriate stimuli, white adipose tissue (WAT) depots can undergo a phenotypic transition toward BAT in a process known as ‘browning,’ resulting in the formation of beige adipose tissue that exhibits characteristics of both depots [79]. In addition, BAT functions as an endocrine organ by releasing signaling molecules that act on multiple peripheral tissues, including WAT, liver, pancreas, heart, and bone, and modulates systemic metabolism through interactions with the central nervous system. These endocrine factors, collectively referred to as ‘batokines,’ include fibroblast growth factor 21 (FGF21), interleukin-6 (IL-6), and neuregulin 4, which exert beneficial metabolic effects and may represent key targets for therapeutic strategies aimed at controlling obesity and its associated metabolic complications [81]. In particular, the interaction between the central nervous system (CNS) and BAT is of considerable relevance, as targeting the brain–BAT axis represents an attractive strategy for combating obesity [94]. However, metabolic adaptations during caloric restriction often attenuate thermogenic output, underscoring the need for further research in this field.

Despite the promising outcomes of IF, it is not devoid of side effects. Limited evidence has documented adverse effects of IF regimens, including hypoglycemia, dizziness, and weakness, with hypoglycemia appearing to be the most severe [147]. Beshyah et al. highlighted a significant risk of hypoglycemia during fasting, particularly in individuals using antidiabetic medications [148]. Accordingly, individuals with diabetes are advised to exercise heightened caution when undertaking IF, as this practice may entail certain risks despite its metabolic benefits. These risks may also extend to individuals with hormonal imbalances, pregnant or breastfeeding women, young children, older adults, and those with immunodeficiencies [64]. Therefore, while IF offers potential benefits, its effects should not be overestimated, and further research is required to confirm its long-term safety in humans.

The limitations of this study include potential publication bias and variability in the quality of evidence across studies. Additionally, most of the clinical trials reviewed involved small sample sizes, and the duration of IF interventions was relatively short, ranging from weeks to months. Further research is warranted regarding molecular mechanisms, ideally focusing on comparative IF regimens in both human and animal models with extended follow-up periods. These studies should also include measurements of batokines and related signaling molecules to directly assess the thermogenic capacity of IF. In the future, large-scale cohort studies are needed to confirm the long-term benefits and potential risks of IF, particularly in individuals with impaired adipose tissue browning or insulin resistance, such as older adults and patients with diabetes. Data generated from these studies could provide deeper insights into the mechanisms underlying the metabolic improvements observed during fasting.

## 8. Conclusions

IF has emerged as a non-pharmacological nutritional strategy with promising effects on body weight regulation, glucose homeostasis, and insulin sensitivity. Current evidence supports its feasibility and metabolic benefits in individuals with obesity or impaired metabolic health; however, its efficacy is closely linked to the physiological mechanisms engaged during fasting and refeeding cycles.

This review highlights that thermogenesis, particularly BAT activation, beige adipocyte recruitment, and CNS–BAT crosstalk, constitutes a central component of the metabolic response to IF. The integration of autonomic, hormonal, nutrient-derived, and circadian signals appears to be essential for coordinating energy expenditure, although important mechanistic gaps remain, including the contribution of UCP1-independent pathways and fasting-induced adaptations in hypothalamic circuits.

The refeeding window also represents a critical opportunity to enhance fasting-induced benefits. Diet-induced thermogenesis, protein quality and timing, fermentable fibers, thermogenic bioactive compounds, and lipid composition emerge as nutritional tools capable of enhancing metabolic flexibility when strategically incorporated into post-fast meals.

IF provides a biologically robust framework to study energy balance and represents a promising avenue for the development of targeted nutritional interventions. Additional research integrating neurobiology, adipose tissue physiology, chrono-nutrition, and multi-omics approaches will be essential to establish evidence-based guidelines and to translate IF into optimized, personalized metabolic therapies.

## Data Availability

No new data were created or analyzed in this study. Data sharing is not applicable to this article.

## Abbreviations

The following abbreviations are used in this manuscript:

IF	Intermittent Fasting
T2DM	Diabetes Mellitus Type 2
T1DM	Diabetes Mellitus Type 1
DIT	Diet-Induced Thermogenesis
CR	Caloric Restriction
ADF	Alternative Day Fasting
PF	Periodic Fasting
TRE	Time-Restricted Eating
CRP	C Reactive Protein
WAT	White Adipose Tissue
BAT	Brown Adipose Tissue
CNS	Central Nervous System
POA	Preoptic Area
DMH	Dorsomedial Hypothalamus
PVH	Paraventricular Nucleus
ARC	Arcuate Nucleus
UCP1	Uncoupling Protein One
